# The Detection of Lung Cancer Cell Profiles in Mediastinal Lymph Nodes Using a Hematological Analyzer and Flow Cytometry Method

**DOI:** 10.3390/cancers17030431

**Published:** 2025-01-27

**Authors:** Iwona Kwiecień, Elżbieta Rutkowska, Agata Raniszewska, Rafał Sokołowski, Joanna Bednarek, Karina Jahnz-Różyk, Piotr Rzepecki

**Affiliations:** 1 Laboratory of Hematology and Flow Cytometry, Department of Internal Medicine and Hematology, Military Institute of Medicine—National Research Institute, 04-141 Warsaw, Poland; erutkowska@wim.mil.pl (E.R.); araniszewska@wim.mil.pl (A.R.); 2 Department of Internal Medicine, Pneumonology, Allergology, Clinical Immunology and Rare Diseases, Military Institute of Medicine—National Research Institute, 04-141 Warsaw, Poland; rsokolowski@wim.mil.pl (R.S.); jbednarek@wim.mil.pl (J.B.); kjrozyk@wim.mil.pl (K.J.-R.); 3 Department of Internal Medicine and Hematology, Military Institute of Medicine—National Research Institute, 04-141 Warsaw, Poland; przepecki@wim.mil.pl

**Keywords:** flow cytometry methods, hematological analyzer, tumor cells, non-small-cell lung cancer, small-cell lung cancer transbronchial ultrasound-guided needle aspiration, epithelial cell adhesion molecule, thyroid transcription factor 1, Ki67, cytokeratin

## Abstract

The presence of metastases in mediastinal lymph nodes (LNs) is essential for planning lung cancer treatment and assessing anticancer immune responses. The aim of the study was to assess LNs for the presence of neoplastic cells and evaluate lung cancer-selected antigen expression. LN aspirates were obtained during an EBUS/TBNA procedure. The cells were analyzed using a hematological analyzer and flow cytometry. It was possible to indicate the presence of cells characterized by high fluorescence connected with high metabolic activity using a hematological analyzer and to determine their non-hematopoietic origin using flow cytometry. Using these methods together, we detected very quickly a high proportion of cancer cells in LNs. We noticed that it was possible to determine a high expression of EpCAM, TTF-1, Ki67, cytokeratin, HER, and differences between non-small-cell (NSCLC) and small-cell lung cancer (SCLC) for the antigens MUC-1, CD56, HLA-DR, CD39, CD184, PD-L1, PD-L2 and CTLA-4 on tumor cells. We report, for the first time, that the detection of tumor cells in LNs with the expression of specific antigens is easy to evaluate using a hematological analyzer and flow cytometry in EBUS/TBNA samples. Such precise characteristics of non-hematopoietic cells in LNs may be of great diagnostic importance in the detection of micrometastases.

## Simple Summary

The study highlights the importance of mediastinal lymph nodes (LNs) in lung cancer treatment and immune response activation. We used EBUS/TBNA to collect LN aspirates and analyzed them via a hematological analyzer and flow cytometry. The hematological analyzer detected highly fluorescent cells with high metabolic activity, while flow cytometry confirmed their non-hematopoietic origin. These combined methods efficiently identified cancer cells in LNs. Key findings include a high expression of markers like EpCAM, TTF-1, Ki67, cytokeratin, and HER, as well as differences between NSCLC and SCLC in antigens such as MUC-1, CD56, HLA-DR, CD39, CD184, PD-L1, PD-L2, and CTLA-4. This study is the first to demonstrate the feasibility of detecting tumor cells and their antigen expression in LNs using these techniques. This precise characterization of non-hematopoietic cells in LNs could significantly aid in identifying micrometastases and refining lung cancer diagnostics.

## 1. Introduction

Lung cancer is one of the most common cancers in the world, and despite advances in our knowledge of diagnosis, risks, immunological management, and treatment options, it remains the leading cause of cancer death [1,2]. Lung cancer is traditionally classified as non-small-cell lung cancer (NSCLC) in 85% of cases and small-cell lung cancer (SCLC) in the remaining 15% [3]. SCLC is highly aggressive in its progression and is most often treated non-surgically, whereas NSCLC is treated with a combination of surgery and adjuvant therapy [4]. The heterogeneity of NSCLC has led to its specific subclassifications into major subtypes, such as adenocarcinoma (ADC), squamous-cell carcinoma (SQCLC), and large-cell carcinoma (LCC) [5]. SCLC is grouped with other tumors that exhibit neuroendocrine differentiation. Detailed histological subtypes are determined based on an analysis of resected specimens to identify tissue types of prognostic significance [6]. Most cases are advanced at diagnosis and inoperable, which complicates treatment and lowers survival rates [7]. As a result, most lung tumors are inaccessible for comprehensive histological diagnostics, and research is incomplete. The small number of resected lung cancer cases results in the very poor accessibility of tumor cells for research In the course of cancer, LNs often become the site of metastases [8], and the assessment of the metastatic status of LN is crucial in predicting the course of the disease [9]. The metastatic LNs are an excellent material for studying cancer cells. The characterization of the LN cell population can be important for the rapid definition of tumor cells before a histopathological analysis.

In the diagnosis of lung cancer, transbronchial ultrasound-guided needle aspiration (EBUS/TBNA) [10] is extremely useful, on the basis of which it is possible to examine enlarged, potentially metastatic mediastinal LNs and infiltrative changes in the lungs. It is known that studies conducted using peripheral blood (PB) do not allow for the demonstration of local changes. The component allowing for obtaining rich cellular material from the direct area of the disease is EBUS/TBNA aspirate. There are a few studies [11,12,13,14], showing the benefits of combining EBUS/TBNA with flow cytometry, which seems to be an accessible, rapid method and the first step in the diagnosis of lung cancer. However, a more advanced multi-antigen panel is needed. The use of 13-color flow cytometry allows for an accurate assessment of the antigenic profile of the tumor cell population. Currently, in the diagnosis of lung cancer, the importance of differentiation not only in the context of the histological type but also concerning the antigenic and molecular profile is emphasized [15].

In the presented work, the selected markers for evaluation using multiparametric flow cytometry are of significant importance in cancer biology: epithelial cell adhesion molecule (EpCAM), Mucin-1 (MUC-1), thyroid transcription factor-1 (TTF-1), human epidermal growth factor receptor 2 (HER-2), cytokeratin, Ki67, CD56, CD90, CD38, HLA-DR, CD39, CD73, CD184, programmed death-ligand 1 (PD-L1), programmed death-ligand 2 (PD-L2), and cytotoxic T cell antigen 4 (CTLA-4). In the literature, markers such as cytokeratin, EpCAM, Ki67, and CD90 allow the confirmation of the presence of cancer cells. Some of the selected antigens are used as diagnostic markers in lung cancer subtypes (TTF-1, MUC-1, HER, cytokeratin, and CD56), others as prognostic, proliferative, or immunosuppressive targets [16,17,18]. Short descriptions of the listed antigens are presented below.

Markers that enable the effective detection of lung cancer cells in mediastinal LNs include cytokeratin [19], which is responsible for the structural integrity of epithelial cells, and EpCAM, a membrane glycoprotein that mediates cell–cell adhesion in the epithelium [20]. Its overexpression is frequently observed in various cancers, including lung, breast, and colorectal cancer, making it a potential target for cancer diagnostics and therapeutic strategies [21,22]. Ki67 is regarded as a marker of proliferation in malignant tumors [20]. Some studies have suggested an association between Ki67 and poor survival in lung cancer patients [23]. CD90 has been described as a potential marker of cancer stem cells (CSCs) and a therapeutic target. CD90-positive CSCs have been associated with high recurrence in some cancers, resistance to multiple chemotherapy drugs, and the occurrence of metastases [24].

Some of the selected antigens may suggest the subtype of the lung tumor or its origin. MUC-1 is a transmembrane glycoprotein that is inappropriately regulated in many types of cancer, including NSCLC, and it plays a key role in the oncogenesis of various human ADCs. Studies have shown that MUC-1 is involved in cell proliferation, invasion, and migration [25] and could be a potential molecule for cancer immunotherapy and targeted therapy [26]. TTF-1 is expressed in approximately 70% of lung ADC, while it is negative for almost all SQCLC in histopathological methods [27]. TTF-1 expression has been also investigated as a prognostic factor in NSCLC with conflicting results [28,29]. Nerve cell adhesion molecule (NCAM), also known as CD56, is a member of the immunoglobulin superfamily and is a recommended marker for the identification of lung tumors with neuroendocrine differentiation [30].

Many markers are being investigated for their usefulness in prognosis or in subsequent targeted therapies. CD38 and HLA-DR are cell surface markers that play important roles in immune response and cancer biology [31]. The potential role of CD38 as a probable biomarker of the immunotherapy response in SCLC is highlighted [32]. HLA-DR, a class II major histocompatibility complex (MHC) molecule, is crucial for antigen presentation and immune surveillance. Its expression can be altered in several cancers, making it a valuable diagnostic and prognostic marker. The expression of CD73 on tumor cells is commonly associated with poor prognosis in several types of cancer, including melanoma, colon cancer, and breast cancer [33]. An interesting association was found between immunohistochemical expression of CD73 in tumor tissues and clinical outcomes in patients with advanced NSCLC [34]. Together with CD73, the ectonucleotidase marker CD39 is widely expressed by lung cancer cells. These molecules induce high extracellular production and release of the immunosuppressive adenosine, which allows for immune escape [35]. CD184 (also known as CXCR4) is a cell surface receptor that, together with the chemokine stromal factor-1 (SDF-1/CXCL12), regulates the migration and metastasis of tumors, including SCLC cells. CD184 expression on SCLC mediates migration, integrin activation, and adhesion to stromal cells [36].

Programmed death receptor-1/programmed death receptor ligands-1 (PD-1/PD-L1/PD-L2) and cytotoxic T-lymphocyte-associated antigen-4 (CTLA-4) or HER are among the most common and promising targets for cancer immunotherapy [37]. PD-L1 and PD-L2 are present on tumor cells, contributing to immunosuppression. The role of the PD-1/PD-L1 pathway has been widely described [38], and the PD-L1 molecule on tumor cells has been validated as a biomarker for immune checkpoint inhibitor (ICI) therapy [39]. Although PD-L2 plays a key role in various human cancers, showing significant potential as a therapeutic target, there is an incomplete understanding of PD-L2 expression and its regulatory mechanism, as well as an insufficient exploration of the related signaling pathways [40]. CTLA-4 is expressed at high levels during T cell activation, where it competes with CD28 for binding to CD80 and CD86 on antigen-presenting cells, sending signals that inhibit T cell activation and their effector functions [41]. A CTLA-4 blockade can inhibit tumor formation, making it useful in treating malignancies 39273605. HER has also emerged in recent years as an interesting therapeutic target for NSCLC. HER-2 changes in NSCLC may include gene mutations, gene amplifications, and the overexpression of the protein on the surface of cancer cells [42].

Currently, lung cancer diagnostics rely on methods that, while accurate, often require significant time and resources, delaying the diagnostic process. To address this gap, we aimed to evaluate whether the combined use of a hematological analyzer and flow cytometry could provide a rapid, reliable, and clinically applicable tool for detecting lung cancer cells in mediastinal LNs.

The aim of this study was to perform an exceptionally fast morphological and flow-cytometric evaluation of metastatic LNs. A detailed antigenic assessment of tumor cells and a combination of methods aimed to propose a new, rapid diagnostic approach for patients with lung cancer, facilitating more efficient diagnostics and enabling the development of additional diagnostic and prognostic markers.

## 2. Materials and Methods

### 2.1. Patients

The study cohort included patients undergoing diagnostic evaluation for lung tumors (n = 64). Of the 64 patients, patients who had received previous or recent anticancer therapy, had clinical signs of infection, had autoimmune diseases, or were undergoing immunosuppressive therapy were excluded from the study. Finally, patients with histologically confirmed primary lung tumors and metastatic lymph nodes (n = 47) were included in the analysis. The classification of tumors followed the current histological guidelines [43]. Written, informed consent was obtained from all participants prior to diagnostic procedures, including EBUS/TBNA, as approved by the Ethics Committee of the Medical Chamber in Warsaw (KB/1441/23).

All patients underwent chest computed tomography before EBUS/TBNA. During the EBUS/TBNA procedure, suspected metastatic lymph nodes were sampled, beginning with the most distal nodal station. Specimens were reviewed by an experienced pathologist to ensure sample quality. Adequate samples were allocated to cytopathological staining, molecular analysis, and flow cytometry.

Lymph nodes were classified as metastatic based on positive cytopathological findings that provided a definitive cancer diagnosis. Samples were collected exclusively during the initial diagnostic evaluation (“point 0”) and were not reused for re-staging. Additionally, as part of routine clinical examinations, molecular diagnostics were performed for some patients. The detailed clinical characteristics of the lung cancer patients are presented in Table 1, with individual data provided in the Supplementary Materials (Table S1).

### 2.2. Materials

Samples from LNs at stations 4R, 4L, 7, 10L, 11R, and 11L were obtained during routine EBUS/TBNA performed for lung cancer diagnostics. After the diagnostic aspiration was completed, an additional aliquot was collected for flow cytometry. Approximately 1 mL of aspirated material was diluted in 0.9% sodium chloride, transferred to K_2_EDTA-containing tubes, analyzed using a hematology analyzer, and subsequently processed for flow-cytometric evaluation. Samples were collected from 1 July 2023 to 1 November 2024 at the Military Institute of Medicine-National Research Institute, Department of Internal Medicine, Pneumonology, Allergology, Clinical Immunology and Rare Diseases, and then transmitted and analyzed at the Laboratory of Hematology and Flow Cytometry, the Department of Internal Medicine and Hematology.

The EBUS/TBNA technique, commonly used in respiratory medicine, enables the collection of material directly from lymph nodes as part of routine diagnostics. This procedure is generally safe, well tolerated by patients, and typically performed without the need for general anesthesia [44]. The LN aspirates obtained through this method are liquid, rich in cellular content, and can be effectively analyzed using flow cytometry or a hematology analyzer.

### 2.3. Analysis Using a Hematology Analyzer

Modern hematology analyzers go beyond standard blood counts by utilizing advanced parameters to evaluate the cellular composition of various liquid samples, providing valuable insights for the preliminary evaluation of disorder conditions. In the study, the Sysmex XN-1500 analyzer (Sysmex Corp., Kobe, Japan) was used to assess the morphology and additional research parameters of LN aspirates. Its operating principle is based on the fluorescence flow cytometry method. The analyzed cells are automatically labeled with a fluorescent dye that binds to nucleic acids. Then, the cells are classified according to the intensity of the FSC light, which indicates the cell volume. The SSC light provides information about the internal structure of the cell and granularity, and the SFL light indicates the amount of DNA and RNA present in the cell. The analyzer also demonstrates the ability to identify a population of cells with high fluorescent activity, forming a specific cloud in the scattergram. This cloud is observed high on the SFL axis (*y*-axis) in the SFL vs. SSC plot, indicating cells with significantly increased metabolic activity. Based on this observation, these cells are considered cells with high metabolic activity and are thought to potentially represent cancer cells.

The Sysmex XN-1500 analyzer provides research parameters that allow for the assessment of the size, complexity, and metabolic activity of neutrophils and lymphocytes in the analyzed samples. The NE-FSC parameter indicates the intensity of forward-scattered light, which indicates the size of neutrophils. The principle of action is based on the amount of bound nucleic acids; the increase in the metabolic activity of neutrophils is accompanied by a higher amount of nucleic acids, which bind fluorescent dye more intensively, leading to an increase in SFL. This is expressed by the parameter NE-SFL (or NEUT-RI), the mean value of fluorescence intensity; it reflects the metabolic activity of neutrophils [45].

In addition, activated neutrophils are characterized by an increase in granularity and the presence of vacuoles, which causes an increase in the SSC value and a change in the position of the neutrophil cloud on the scattergram. This is reflected in the change in the value of the parameter NE-SSC (or NEUT-GI) expressed as scattering intensity. NE-SSC provides information about the density or complexity of the cell and depicts the granularity of the cells [45,46].

**Figure 1:**
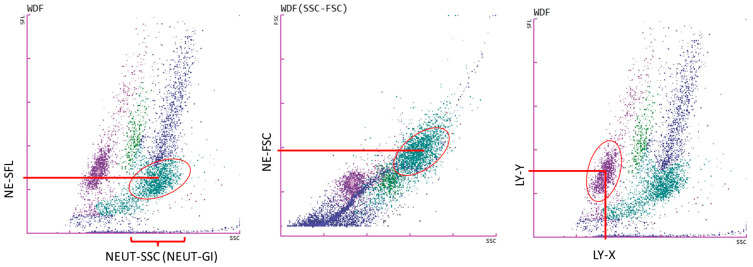
The graphs show the white blood cell differential channel (WDF) for neutrophils (first two graphs from the left) and for lymphocytes (last graph on the right). The first scattergram of neutrophils shows a scattergram dispersion value (NEUT-SSC) on the *x*-axis and the median scattergram value of the fluorescence side (NE-SFL) on the *y*-axis. The middle one shows the median scattergram value of the forward scattergram (NE-FSC) on the *y*-axis. Right: lymphocyte positional parameters scattergram showing the median scattergram value (LY-X) on the *x*-axis and the median scattergram value of the fluorescence side (LY-Y) on the *y*-axis.

The Sysmex XN-1500 analyzer also enables the evaluation of lymphocyte-specific parameters, which allows a detailed characterization of lymphocyte morphology and functional status in analyzed samples. The LY-X parameter represents the intensity of laterally scattered light, providing information about the complexity of lymphocytes, including their internal structure and granularity. The LY-Y parameter reflects the intensity of fluorescent light, which correlates with the fluorescence of lymphocytes, offering insights into their metabolic activity and nucleic acid content [47,48]. The neutrophil and lymphocyte positioning parameters on the Sysmex XN analyzer are presented in Figure 1.

### 2.4. Flow-Cytometric Analyses

The percentage composition of LNs of aspirates was assessed via multiparameter flow cytometry with a panel of monoclonal antibodies, using DxFLEX flow cytometry (Beckman Coulter Company, Marseille Cedex 9, France). The flow cytometer undergoes daily quality control for correct operation within the specified parameters. Calibration beads are used to check the analyzer configuration, measure the power of each laser, and calibrate the signal gain setting. Cells of hematopoietic and non-hematopoietic origin were distinguished. The following monoclonal antibodies were used to assess the cells’ composition:

- CD64-FITC (catalog number: 555527, clone number: 10.1 RUO, BD Biosciences, Herlev, Denmark);
- Fibroblasts Monoclonal Antibody (FMA)-PE (catalog number: MA5 16642, clone number: D7-FIB, Invitrogen, Carlsbad, CA, USA);
- CD146-PE-Cy7 (catalog number: 562135, clone number: P1H12, BD Biosciences, Herlev, Denmark);
- CD19-PE-DyLight 594 (catalog number: AQ335127, clone number: LT19, Sysmex, Norderstedt, Germany);
- CD3-PC-5.5 (catalog number: B49203, clone number: UCHT1, Beckman Coulter);
- CD8-APC (catalog number: IM2469, clone number: B9.11, Beckman Coulter);
- CD326 (Ep-CAM)-AF700 (catalog number: 324244, clone number: 9C4);
- CD16-APC-H7 (catalog number: 560195, clone number: 3G8, BD Biosciences, Herlev, Denmark);
- HLA-DR-V450 (catalog number: 655874, clone number: L243, BD Biosciences, Herlev, Denmark);
- CD45-V500 (catalog number: 655873, clone number: 2D1, BD Biosciences, Herlev, Denmark);
- CD4-BV650 (catalog number: 300536, clone number: RPA-T4, BioLegend, San Diego, CA, USA).

Next, the cells were distinguished based on their antigenic properties: lymphocytes (CD45+ SSC-A+dim), lymphocytes T (CD45+ CD3+ SSC-A+dim), CD4+ T lymphocytes (CD45+ CD3+ SSC-A+dim CD4+), CD8+ T lymphocytes (CD45+ CD3+ SSC-A+dim CD8+), natural killer (NK) cells (CD45+ CD3- SSC+dim CD16+), neutrophils (CD45+ CD3- SSC-A+bright CD16+), monocytes (CD45+ CD64+ HLA-DR+ SSC-A+), an endothelium (CD45- CD146+ SSC-A+), a fibroblast (CD45+dim FMA+ SCC-A+), and probably tumor cells (CD45- EpCAM+ SSC-A+brigh CD146- FMA-). The gating strategy for the analyzed cells is presented in the Supplementary Materials (Figure S1).

Then, the expression of selected antigens on cancer cells was analyzed to more precisely define the origin of these cells. We used the following antibodies for this purpose:

- CD326 (EpCAM)-BV 605 (catalog number: 324224, clone number: 9C4, BioLegend);
- MUC-1-APC (catalog number: 355608, clone number: 16A, BioLegend);
- TTF-1-Alexa Fluor 700 (catalog number: NBP3-21041AF700, clone number: 8143R, NovusBio, Centennial, CO, USA);
- Ki67-FITC (catalog number: F7268, clone number: MIB-1, Dako);
- Cytokeratin-PE (catalog number: 347204, clone number: CAM 5.2, BD Biosciences);
- CD56-ECD (catalog number: B49214, clone number: N901, Beckman Coulter);
- CD38-ECD (catalog number: C86903, clone number: LS198-4-3, Beckman Coulter);
- HLA-DR-BV 605 (catalog number: 307640, clone number: L243, BioLegend);
- HER-2-PE (catalog number: 340552, clone number: Neu 24.7, BD Biosciences);
- CD39-PerCP-Cy 5.5 (catalog number: 564899, clone number: TU66, BD Biosciences);
- CD73-BV785 (catalog number: 344028, clone number: AD2, BioLegend);
- CD90-PE Cy-7 (catalog number: 561558, clone number: 5E10, BD Biosciences);
- CD184-APC (catalog number: 555976, clone number: 12G5, BD Biosciences);
- PD-L1-PE (catalog number: 557924, clone number: MIH1, BD Biosciences);
- PD-L2-BV421 (catalog number: 563842, clone number: MIH18, BD Biosciences);
- CD152 (CTLA-4)-PE-Cy 5 (catalog number: 555854, clone number: BNI3, BD Biosciences);

A cell viability test was performed for each sample using trypan blue and the 7-AAD reagent. More than 95% of viability was achieved. For each sample, a minimum of 300,000 events were collected. Based on the experience of a previous flow cytometry study, the Fluorescence Minus One (FMO) control for the characteristics of selected antibodies and isotype control was used to detect tumor cells and to eliminate the high autofluorescence of these cells. The method of gating tumor cells is described in Section 3.3. The cellular characterization of lymph nodes was performed using flow cytometry methods.

### 2.5. Statistic Analysis

All statistical analyses were performed using the Statistica 13.0 software (TIBCO Software, Palo Alto, CA, USA). The Shapiro–Wilk test was used to assess the assumption of normal distribution. Since the analyzed parameters did not meet the criteria for a normal distribution, the nonparametric Mann–Whitney U test was employed to compare two groups (NSCLC patients and SCLC). The results are presented as means with standard deviations (SDs) or medians with interquartile ranges (Q1–Q3). Statistical significance was considered at *p* < 0.05. The GraphPad Prism (version 7, GraphPad Software, La Jolla, CA, USA) and Kaluza C Analysis Software (version 1.1, Beckman Coulter Software, Marseille Cedex 9, France) were used for graphical processing.

## 3. Results

### 3.1. Patients’ Clinical Characteristics

The study group consisted of 47 patients with confirmed lung cancer (female = 24, men = 30; mean age: 68.1 ± 8.4). Most patients in the study group were in an advanced stage of lung cancer (stage I: 2, 2.7%; II: 3, 1.4%; III: n = 22, 46.8%; and IV: n = 20, 42.6%). We divided the group to SCLC patients (n = 27) and NSCLC patients (n = 20). Among patients with NSCLC, the highest incidence was observed for adenocarcinoma (ADC) (n = 9, 45.0%), followed by squamous-cell lung cancer (SQCLC) (n = 6, 30.0%) and next for not otherwise specified (NOS) (n = 4, 20.0%). Due to the small number of patients, we did not perform a comparison between groups with different types of NSCLC. The characteristics of the groups are presented in Table 1, and additionally, in the Supplementary Materials, the results for individual patients with regard to cancer subtype and molecular results are presented.

### 3.2. Cells Analysis of Lymph Node Aspirates Using a Hematological Analyzer

**Figure 2:**
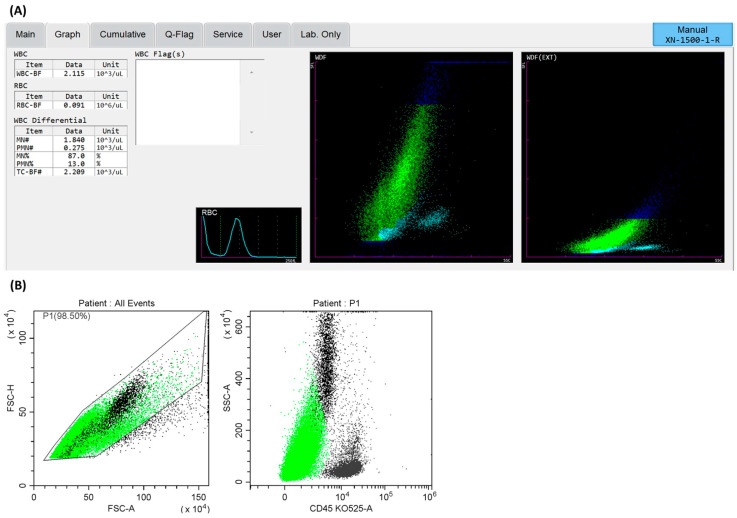
Dot plots with cells (probably cancer cells—green cells) from lymph node aspirates. (**A**) Dot plot of SSC vs. SFL for the WDF and WDF (EXT) channels on a Sysmex XN analyzer, showing the distribution of high-fluorescence (SFL high) cells in the lymph node aspirates. (**B**) Flow cytometer dot plots: FSC-A vs. FSC-H plot, gating the cells and thus removing clumps (greater FSC-A relative to FSC-H) and debris (very low FSC), CD45, vs. SSC-A plot, showing cell distribution against the presence of the CD45 antigen and the side-scatter (SSC) parameter (probably cancer cells: CD45- SSC+bright).

We found that, in the majority of the tested LN aspirates, according to a hematological analyzer, the predominant population was probably tumor cells. It was possible to indicate the presence of cells usually characterized by high fluorescence (Figure 2A). This suggests their high metabolic activity. The cellularity measured using a hematology analyzer was sufficient for flow-cytometric analysis and was, on average, 3325 ± 4855.3 cells.

In the next step, we demonstrated that, with the 13-color cytometry method with a panel of monoclonal antibodies (a detailed description of the monoclonal antibodies used can be found in the Methodology section’s subsection on flow cytometry methods), it is possible to determine their non-hematopoietic origin.

### 3.3. Cellular Characterization of Lymph Nodes via Flow Cytometry Methods

We focused on the cellular characteristics of LN by assessing hematopoietic cells (lymphocytes and lymphocyte subpopulations: T and B lymphocytes, NK cells, then neutrophils, monocytes, and dendritic cells) and non-hematopoietic cells (endothelium and fibroblasts) at the site of direct disease development, separating them from cells with the CD45- phenotype, characterized by high fluorescence (side-scatter SSC+bright) (Figure 2B). We found that, in most of the examined material, the dominant population was probably neoplastic cells, showing no expression of the CD45 antigen or high fluorescence and, via the elimination path, not being endothelia or fibroblasts. The remaining subpopulations were cells of hematological origin (in small numbers, significantly lower than cancer cells, neutrophils and lymphocytes dominated among cells of hematopoietic origin). Additionally, small numbers of endothelial cells and fibroblasts were found (Table 2).

### 3.4. Antigenic Profile of Tumor Cells in Metastatic Lymph Nodes

**Figure 3:**
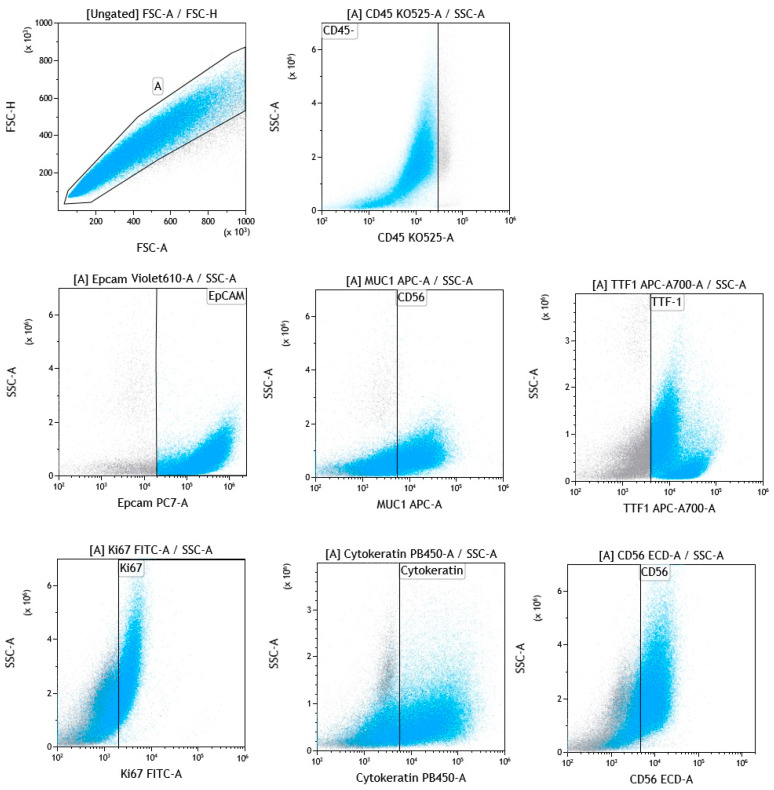
Expression of selected antigens (EpCAM, MUC-1, TTF-1, Ki67, cytokeratin, and CD56) on CD45- cells (cancer cells) from EBUS/TBNA aspirates via the flow cytometry method in selected metastatic LNs.

**Figure 4:**
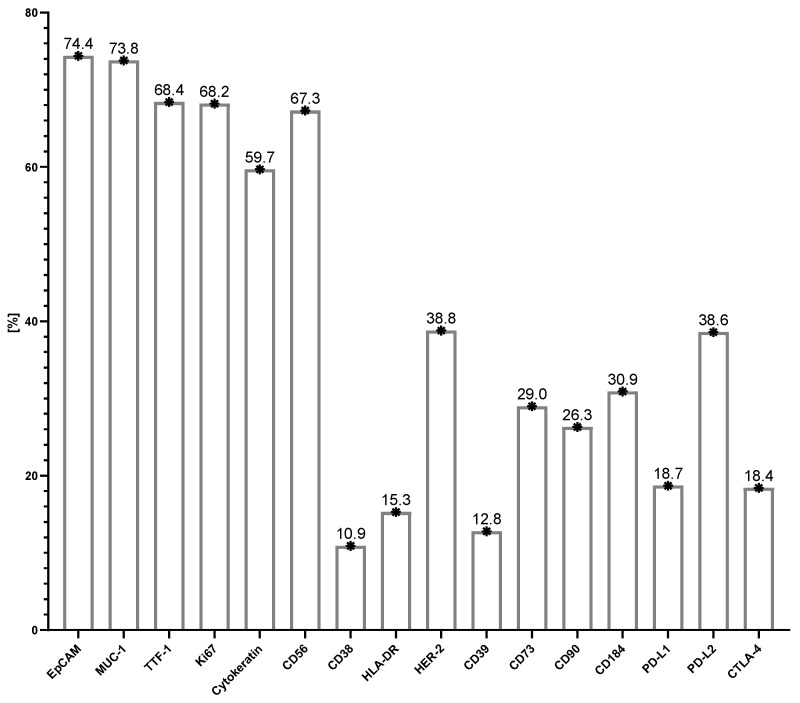
Characteristics of the study group with lung cancer in terms of the relative expression of selected antigens in affected lymph nodes (* arithmetic mean of % antigen expression). Abbreviations: EpCAM—epithelial cell adhesion molecule; MUC-1—Mucin-1; TTF-1—thyroid transcription factor-1; HER—human epidermal growth factor receptor; PD-L1—Programmed death-ligand 1—PD-L2: Programmed death-ligand 2; CTLA-4—cytotoxic T cell antigen 4.

**Figure 5:**
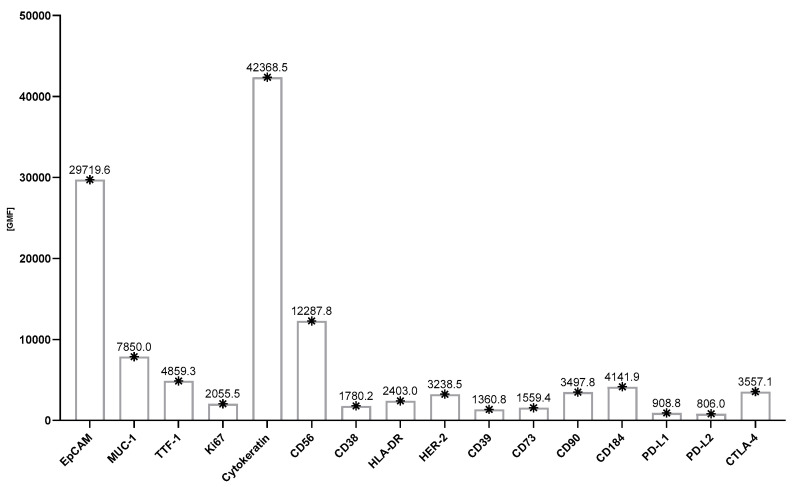
Characteristics of the study group with lung cancer in terms of the relative expression of selected antigens in affected lymph nodes (* arithmetic mean of GMF: geometric mean fluorescence of antigen expression). Abbreviations: EpCAM—epithelial cell adhesion molecule; MUC-1—Mucin-1; TTF-1—thyroid transcription factor-1; HER—human epidermal growth factor receptor; PD-L1—Programmed death-ligand 1; PD-L2—Programmed death-ligand 2; CTLA-4—cytotoxic T cell antigen 4; GMF—geometric mean fluorescence.

The use of 13-color flow cytometry in the study allowed for a precise assessment of the antigen profile of the above-mentioned cancer cells in LN aspirates. The dominant antigens on cancer cells were EpCAM, MUC-1, TTF-1, Ki67, cytokeratin, and CD56, whose average expression was around 70–60%. These selected antigens are presented in Figure 3. A moderately high expression was also observed for HER-2 and PD-L2 molecules on cancer cells (approximately 40%). A lower expression was observed for the following molecules: CD38, HLA-DR, CD39, CD73, CD90, CD184, PD-L1, and CTLA-4 on cancer cells (the average value of which was around 10–30%) (Figure 4). When the GMF of the tested antigens’ expression was considered, the highest value was observed for EpCAM and cytokeratin molecules (Figure 5).

### 3.5. Differences in the Antigen Profile of Tumor Cells, Depending on the Type of Cancer

We analyzed the differences in LNs’ cellular composition and expression of molecules found on tumor cells, depending on the histological subtype of cancer.

We compared the percentage composition of cells in the LNs of patients with NSCLC and SCLC. The patients with NSCLC constituted a statistically higher proportion of lymphocytes (respectively, 8.5 vs. 4.0%, *p* = 0.0249), lymphocytes T (respectively, 6.3 vs. 3.2%, *p* = 0.0296), lymphocytes B (1.8 vs. 0.6%, *p* = 0.0331), and fibroblasts (respectively, 1.2 vs. 0.4%, *p* = 0.0313) than that of patients with SCLC. The tumor cells’ proportion and the number of events found in the flow cytometer were lower for NSCLC patients than SCLC patients (respectively, 62.5 vs. 88.5%, *p* = 0.0127, and 13792 vs. 50829 events, *p* = 0.0350) (Table 3).

**Figure 6:**
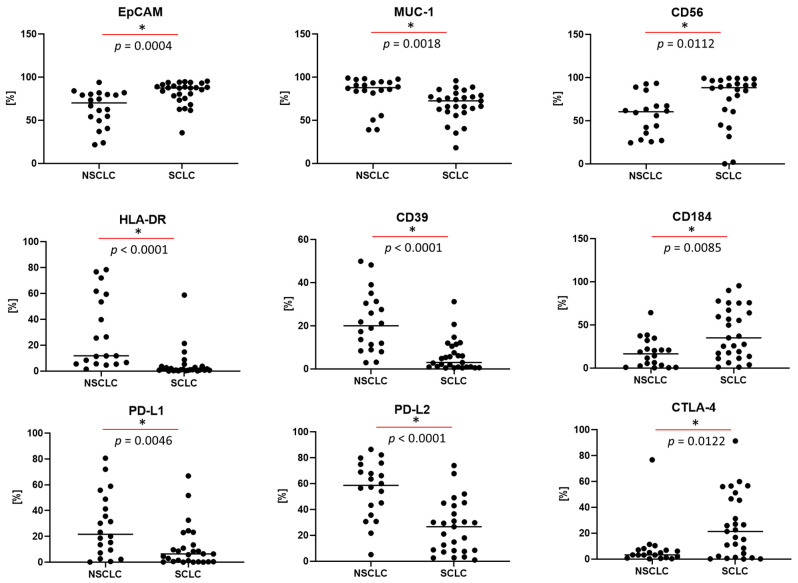
The differences in the proportion of selected antigens (statistically significant) between patients with non-small-cell lung cancer (NSCLC) and those with small-cell lung cancer (SCLC). Data expressed as medians. * indicates statistically significant *p* < 0.05.

Next, we analyzed the differences in the tested molecules on tumor cells in LN aspirates from patients with NSCLC and SCLC. We observed a statistically lower proportion of EpCAM and CD56 expression on tumor cells in patients with NSCLC than those with SCLC (respectively, 70.1 vs. 87.5%, *p* = 0.0004, and 60.4 vs. 88.5%, *p* = 0.0112). The proportion of MUC-1 expression was statistically higher in NSCLC patients than SCLC patients (respectively, 88.0 vs. 72.6, *p* = 0.018). Patients with NSCLC had a statistically higher proportion of HLA-DR and CD39 expression than patients with SCLC (respectively, 11.8 vs. 1.1, *p* < 0.0001, and 20.0 vs. 3.0, *p* < 0.0001) and a statistically lower proportion of CD184 expression than patients with SCLC (16.5 vs. 35.1%, *p* = 0.0085). When the expression of checkpoint antigens on cancer cells was considered, a statistically higher proportion of PD-L1 and PD-L2 expression was demonstrated in NSCLC patients than in SCLC patients (respectively, 21.6 vs. 6.4%, *p* = 0.046, and 58.7 vs. 26.7%, *p* < 0.0001), as well as a statistically lower proportion of CTLA-4 expression in NSCLC patients than in SCLC patients (respectively, 3.2 vs. 21.4%, *p* = 0.0122) (Figure 6, Table 4). The same differences were observed when the GMF values of the tested antigens were considered. No differences were observed for antigens highly expressed in cancer cells, i.e., TTF1, Ki67, and cytokeratin.

Differences were also demonstrated in additional hematological parameters concerning lymphocyte and neutrophil complexity (Table 5). We observed a statistically higher proportion of the NE-SSC parameter in patients with NSCLC than in SCLC patients (respectively, 150.6 vs. 119.1, *p* = 0.0296) and a statistically lower proportion of the LY-X parameter in patients with NSCLC than in SCLC patients (respectively, 83.9 vs. 95.0, *p* = 0.0075).

## 4. Discussion

The study included patients with confirmed lung cancer, and the patients were divided into SCLC (n = 27) and NSCLC (n = 20) groups. Globally, approximately 80–85% of all lung cancer cases are NSCLC, whereas SCLC typically accounts for approximately 10–15% of cases. This division reflects differences in biology, risk factors, and clinical behavior between the two subtypes [49,50]. Within the NSCLC subgroup, ADC was the most common histological subtype. The predominance of ADC among NSCLC patients in our cohort is consistent with global trends, as this subtype has been increasingly observed to be the most frequent form of NSCLC [51,52]. Similarly, the representation of SQCLC reflects its recognized contribution to NSCLC cases, albeit to a smaller extent than ADC. Detailed patient-by-patient data are included in the publication (Supplemental Table S1), providing valuable insights into the heterogeneity across cancer subgroups including NSCLC and the diversity of molecular features. These data underscore the importance of personalized approaches in lung cancer research and clinical decision-making. However, the relatively small sample size in our study precluded comparisons between different NSCLC subtypes, limiting the generalizability of findings related to these two specific groups. Further studies with larger cohorts are warranted to investigate subtype-specific differences and their implications for diagnostic and therapeutic strategies.

In this study, we explored the cellular composition of LN aspirates in lung cancer patients using a hematological analyzer, which revealed that the majority of the identified cells exhibited characteristics commonly associated with tumor cells. These cells were distinguished by their high fluorescence, suggesting increased metabolic activity. This finding underscores the potential utility of hematological analyzers in rapidly detecting and quantifying tumor cells in LN samples. There is a lack of reports on this subject. In the literature, highly fluorescent cells forming a characteristic cloud on a hematology analyzer are used to evaluate leukemic cells and reactive lymphocytes in the context of hematological disorders and infections [53,54,55]. These are usually works that combine the use of a hematology analyzer with other methods; e.g., Eilertsen H. et al. evaluated the utility of blast detection using Sysmex hematology devices, CellaVision DM96, and manual microscopy with flow cytometry as a confirmatory method. In the literature, there are some works on the use of the high fluorescence (HF) cell parameters from a hematology analyzer to evaluate malignant cells [56]. The malignant samples had significantly higher HF counts compared to nonmalignant samples. The study concluded that the HF count is a valuable screening tool for detecting malignant cells in serous fluids, aiding in the selection of samples for further examination [57]. Studies have shown that the analyzer’s ability to identify abnormal cells using the WDF and WDF (EXT) scattergram patterns is useful for confirming the presence of tumor cells, including those in serous fluids [55,58,59]. These findings highlight the potential for integrating Sysmex analyzers in clinical settings as a screening tool, complementing traditional cytological examination.

In our study, a further confirmation of the presence of tumor cells and their non-hematopoietic origin was obtained using 13-color flow cytometry with a panel of monoclonal antibodies. This approach appears to increase the diagnostic precision for identifying metastatic tumor cells in LNs, providing key information on disease progression and a further antigen profile. We conducted an analysis of LN aspirates using flow cytometry to assess both the cellular composition and antigen expression profiles, focusing on potential cancer cells. One of the most common sites of the metastatic spread of lung cancer is mediastinal LNs [60]. In recent years, EBUS-TBNA has proven to be an excellent, minimally invasive tool for assessing changes in LNs in an outpatient setting. Many studies have shown that it is at least as accurate as mediastinoscopy and has fewer complications [61,62]. The study by Yamamoto S et al. [63] highlights the effectiveness of EBUS-TBNA in lung cancer diagnosing and assessing mediastinal/hilar lymphadenopathy. It emphasizes its minimally invasive nature, high sensitivity, and utility in distinguishing benign from malignant lesions, contributing significantly to accurate staging and treatment planning in lung cancer patients.

Initially, in our study, we identified a population of CD45-high fluorescent cells (SSC+bright) that predominated in most LN samples, which we assumed to be neoplastic cells. These cells were distinguished from non-hematopoietic and hematopoietic cells by the lack of CD45 expression and their exclusion from endothelial or fibroblast lineages. For confirmation that these cells were of neoplastic origin, we used a panel of monoclonal antibodies to assess their antigenic profile. This analysis revealed the strong expression of epithelial cell markers such as EpCAM and MUC-1 (approximately 70–60%), along with other cancer-associated markers, including TTF-1, Ki67, cytokeratin, and CD56. Moderate expression was observed for HER-2 and PD-L2 (approximately 40%), whereas lower expression was noted for molecules such as CD38, HLA-DR, CD39, CD73, CD90, CD184, PD-L1, and CTLA-4 (approximately 10–30%). Among these markers, the highest mean GMF levels were observed for EpCAM and cytokeratin, which are well-known markers of epithelial malignancies, further supporting the presence of tumor cells in LNs. A review of the literature did not reveal any studies evaluating such a broad panel of antitumor antibodies in a single LN aspirate sample using flow cytometry as described in our study. Most current research focuses on fewer markers or relies on more traditional diagnostic techniques. For example, flow cytometry has been used to detect specific markers in lung cancer, but in the context of a single marker or in combination with other diagnostic tools such as histopathology or PCR-based methods. The study by Gostomczyk K et al. [64] compared flow cytometry and cytology for the detection of malignant cells in peritoneal and pleural fluids, using antibodies against CD45, CD14, and EpCAM to identify malignant cells. Flow cytometry showed a significantly higher sensitivity compared to cytology for the detection of malignant cells, while both methods showed equal specificity. However, in this study, the detection of malignant cells was based on a limited number of markers, focusing mainly on EpCAM expression. Bugalho A. et al. evaluated the use of EBUS-TBNA combined with flow cytometry and reverse-transcription quantitative polymerase chain reaction (RT-qPCR) to identify tumor-associated antigens: EpCAM, cytokeratin-19 (CK-19), carcinoembryonic antigen (CEA), and CD44 in patients with NSCLC. They found an increased expression of CK-19, CEA, and EpCAM in NSCLC samples than in control samples. The study suggests that the detection of CK-19, CEA, and EpCAM through these methods may improve the detection of LN metastases in NSCLC [20]. San W. et al. [65] evaluated the diagnostic utility of MUC-1 and EpCAM mRNA as tumor markers to distinguish benign from malignant pleural effusions. The authors showed that these markers exhibit high sensitivity and specificity, making them reliable tools for diagnostic purposes. Wang C. et al. [66], using lung cancer cell lines, reported high MUC-1 expression in most of the lines and variable levels of EpCAM, emphasizing their diagnostic and therapeutic potential in lung cancers. In our study, the expression of MUC-1 antigen was also high, the second highest after EpCAM. In other works, researchers focus on the evaluation of EpCAM+ CD45+ cells both in tumor tissues and in the peripheral blood of patients with lung cancer. This contrasts with our work, in which we showed that all analyzed tumor cells were CD45-. Furthermore, EpCAM expression correlated with serum tumor markers such as CEA, which increases its importance in cancer detection and monitoring [67].

The next markers found in significant amounts on tumor cells in our study were as follows: TTF-1, cytokeratin, Ki67, and CD56. The high expression of these antigens on tumor cells is consistent with data from the literature. TTF-1 is a well-known marker in the diagnosis of lung cancer, especially in ADC and SCLC. It is mainly assessed using immunohistochemistry (IHC) because it provides nuclear staining that is highly specific for lung and thyroid cancers. IHC studies have shown TTF-1 positivity in 60–90% of lung adenocarcinoma cases and 82% of SCLC cases, making it a key diagnostic and prognostic tool [68]. There is emerging interest in using flow cytometry to assess its expression. This method could potentially provide a more quantitative and scalable approach to the analysis of protein expression in tumor cells. However, there is currently a lack of widespread documentation of TTF-1 assessment via flow cytometry in the literature, suggesting that this area is underexplored and may represent a new opportunity for research. We were unable to find studies that assessed cytokeratin expression in combination with other tumor markers via flow cytometry. Most studies rely on the use of IHC [69], with some new approaches integrating cytometry for broader cell-type profiling. The study by Lemieux, M.E. et al. [70] examined the early detection of lung cancer by combining automated flow cytometry with machine learning models. The researchers used epithelial markers such as EpCAM and cytokeratin to identify malignant cells in sputum samples and combined this with machine learning to improve diagnostic accuracy. Flow cytometry data were integrated with computational algorithms for automated cell classification and pattern analysis, enabling the robust detection of malignant cells.

While IHC remains the standard method for assessing the Ki67 antigen as well, flow cytometry is emerging as an accurate alternative, especially for quantifying proliferative activity. High Ki-67 expression is associated with rapid tumor growth, indicating its importance as a prognostic marker [71]. Our study is consistent with the literature data, confirming high Ki67 expression in most of the analyzed samples (the mean expression was 68.2%).

CD56 expression helps distinguish SCLC from other lung cancer subtypes. Studies using IHC and flow cytometry have shown that CD56 is a critical marker for characterizing neuroendocrine tumors, which has diagnostic and potential therapeutic implications [72,73]. In our study, the average expression of this antigen on tumor cells was clear and amounted to 67.3%.

The antigens discussed above, including EpCAM, MUC-1, TTF-1, Ki67, cytokeratin, and CD56, which showed the highest and most consistent expression on tumor cells, may constitute a stable and reliable panel of markers for assessing the presence of lung cancer cells in the rapid diagnostic process. In our group, the other tested markers show a lower expression on cancer cells; however, this does not exclude their potential role in personalized diagnostics. Scientific reports, as mentioned below, suggest their significance in the context of lung cancer, indicating the possibility of their application in selected patients.

Examined marker HER-2 is a critical and therapeutic target, especially in breast, gastric, and lung cancer, mainly assessed via IHC and fluorescence in situ hybridization to determine the degree of tumor aggressiveness. These assessments are crucial for HER-2-targeted therapies, such as trastuzumab [74,75]. Wopereis S. et al. [76] focused on the use of flow cytometry to analyze HER2 expression in various breast cancer cell lines. Combining flow cytometry with other molecular tests has been shown to increase the diagnostic accuracy of HER2-positive tumors. Although flow cytometry is not commonly used to assess HER-2, our study highlighted its potential to quantify HER-2 on tumor cells in LNs.

In the next stage of our research, we showed a difference in the tested antigens on tumor cells between NSCLC and SCLC. Interestingly, the patients differed in terms of cellular distribution. In patients with SCLC, we showed a statistically significantly higher percentage of tumor cells in LNs than in LNs of NSCLC. SCLC tends to grow faster and metastasize earlier than NSCLC [77]. SCLC is characterized by a high proliferative capacity and rapid growth, leading to the rapid spread of cancer to LNs and other organs [78]. Although NSCLC can also metastasize, it usually grows slower, allowing more time for potential treatment options [52]. Furthermore, differences in antigen expression were observed between NSCLC and SCLC in our study. NSCLC showed higher proportions of MUC-1, HLA-DR, and CD39 expression, while SCLC had higher levels of CD56, EpCAM, and CD184. No significant differences were found in the expression of TTF-1, Ki67, or cytokeratin or CD73, CD90, and CD38 in both types of cancer. Higher expression of the MUC-1 antigen in NSCLC may be related to the fact that it is a marker of lung adenocarcinoma, which is supported by histopathological studies [79]. CD56 assessment is useful in the diagnosis of SCLC and other neuroendocrine tumors, which explains the higher expression of this marker in SCLC than in NSCLC [80]. In our study, we also observed a higher expression of CD39 and HLA-DR antigens in NSCLC than in SCLC. CD39 expression in NSCLC cells is an important area of research due to its role in the TME. Studies have shown that CD39 is upregulated in various cell types in NSCLC tumors, including NK cells, fibroblasts, and T cells [81]. A low percentage of CD39-positive cells was observed in NSCLC cell lines [82]. CD39 contributes to the immunosuppressive effect on tumor microenvironments by converting extracellular ATP to adenosine [83]. It is hypothesized that this mechanism may play a significant role in EGFR-positive NSCLC [84]. However, more research is needed to support this hypothesis. Studies have shown that the HLA-DR marker presents tumor-associated antigens (TAAs) that are recognized by CD4+ T cells, which then produce cytokines to inhibit tumor growth [85]. Mei et al. found that HLA-DR expression was associated with an inflammatory TME and identified immuno-hot tumors in NSCLC [86]. They also found that HLA-DR was associated with multiple pathways related to immunotherapy. In SCLC, we observed a significantly higher expression of the CD184 antigen, which is consistent with the observation of other investigators. CD184 is highly expressed in SCLC cells, and its interaction with its ligand, CXCL12, promotes tumor growth and metastasis [78]. Our findings suggest distinct immunologic profiles in NSCLC and SCLC and are consistent with the results of other studies suggesting a fundamentally different biology in the two types.

NSCLC also showed a higher expression of PD-L1 and PD-L2 and lower levels of CTLA-4 compared to SCLC. In SCLC, a higher expression of CTLA-4 suggests a mechanism of immune evasion via the inhibition of the checkpoint receptor on T cells. The aggressive neuroendocrine tumor biology of SCLC may increase CTLA-4 expression as a means of suppressing T cell-mediated immunity [87]. In contrast, NSCLC tumors show higher levels of PD-L1 and PD-L2 promoting immune tolerance, which may be particularly important in the more heterogeneous, immunosuppressive microenvironment of NSCLC [88]. CTLA-4 expression may vary across different stages of lung cancer, including NSCLC and SCLC. In the early stages of lung cancer, CTLA-4 expression may be lower because the immune system is still actively trying to fight the cancer cells [89]. However, as the cancer progresses, CTLA-4 expression may increase, contributing to immune evasion by the cancer cells. Higher levels of CTLA-4 expression are often seen in more advanced stages of lung cancer [90]. Such differences underscore that immune checkpoint blockade therapies may require different tailoring for NSCLC than for SCLC, with PD-1/PD-L1 inhibitors potentially being more effective in NSCLC, whereas CTLA-4 inhibitors may better address immune suppression in SCLC.

Our study also highlights important differences in the hematological profiles between patients with NSCLC and SCLC, particularly with respect to the parameters NE-SSC and LY-X. Elevated NE-SSC values in patients with NSCLC reflect increased neutrophil complexity, possibly related to increased activation and granularity, characteristic of systemic inflammatory reactions. On the other hand, higher LY-X values in patients with SCLC suggest increased lymphocyte complexity, potentially reflecting immune dysregulation and altered lymphocyte populations. These findings underscore distinct immunologic and inflammatory landscapes in these lung cancer subtypes, which may reflect their different tumor microenvironments and systemic responses. The observed higher NE-SSC in NSCLC patients is consistent with the results of other studies that associate increased NE-SSC with increased neutrophil activation in inflammatory conditions [91,92]. The lymphocyte complexity observed in SCLC patients, indicated by increased LY-X values, is consistent with the patterns of immune dysregulation described in the literature [93] and other works highlighting lymphocyte changes in inflammatory and immunocompromised conditions [47]. These changes may reflect a more profound effect of SCLC on adaptive immunity compared to NSCLC. The evaluation of NE-SSC and LY-X parameters in LN aspirates from lung cancer patients may provide information on tumor-related immune and inflammatory responses at the microenvironmental level, opening new avenues of research and potential biomarkers.

Our approach, using a broad antibody panel for the fast antigenic profiling of tumor cells in LN samples, combined with the high-resolution capabilities of flow cytometry, appears to be novel in the context of lung cancer diagnosis. Although flow cytometry itself is not a new method, its application in this comprehensive manner to rapidly assess multiple markers in tumor LNs, together with screening via a hematology analyzer, represents a novel approach. In this manuscript, we have provided a comprehensive review of the current state of research regarding the assessment of lymph nodes (LNs), particularly in the scientific context. To date, there are no studies in the literature that demonstrate the use of flow cytometry for cancer-affected LNs in clinical diagnostics. As emphasized, currently, lung cancer diagnosis predominantly relies on imaging techniques such as computed tomography (CT) or positron emission tomography (PET) scans, followed by a histopathological evaluation of LNs. This highlights the novelty of our approach, which combines flow cytometry and a hematological analyzer for the rapid analysis of LN aspirates, a method not previously described in the literature.

## 5. Conclusions

The results of this study provide new evidence supporting the effectiveness of combining a hematological analyzer and flow cytometry in assessing LN aspirates obtained during the EBUS/TBNA procedure. For the first time, we demonstrated the ability to rapidly detect tumor cells with high fluorescence and metabolic activity and determine their non-hematopoietic origin in LN samples. The findings also enable a detailed characterization of the expression of selected cancer antigens, which is of significant importance in differentiating between NSCLC and SCLC. Our study highlights the potential of the hematological analyzer combined with flow cytometry as a rapid and precise diagnostic tool for detecting metastases in LNs. The precise characterization of non-hematopoietic cells in LNs, with attention to specific antigen expression, may play a key role in improving lung cancer diagnostics.

## Figures and Tables

**Table 1:** Patient characteristics.

	All Studies Groups	
Number of patients	47	
Sex f/m (n)	24/23	
Age (mean ± SD years)	68.1 ± 8.4	
LNs: 4R/4L/7/10R/10L/11R/11L/ *	12/7/9/0/1/1/1/4/12	
Stage: I/II/III/IV (n)	2/3/22/20	
Tumor cells % (mean ± SD years)	70.4% ± 25.1	
Tumor cells events (mean ± SD years)	66,300 ± 107,839	
Histological types:		
	NSCLC	SCLC
Number of patients	20	27
Age (mean ± SD years)	69.2 ± 8.8	67.3 ± 8.2
Sex f/m (n)	10/10	14/13
Histological subtypes:		
SQCLC	6	n/a
ADC	9	n/a
NOS	4	n/a
LCC	1	n/a

Abbreviations: f—female; m—male; n—number; LNs—lymph nodes; NSCLC—non-small-cell carcinoma; SCLC—small-cell carcinoma; SQCLC—squamous-cell lung carcinoma; ADC—lung adenocarcinoma; NOS—not otherwise specified; LCC—large-cell carcinoma; *—tumor mass, n/a—not applicable.

**Table 2:** Cells proportion in lymph node aspirates (LNs) of patients with lung cancer. Data expressed as means and standard deviations (SDs).

Leukocytes Subpopulation and Tumor Cells: (via Flow Cytometry Methods) [% of All Cells]	Lung Cancer Mean ± SD
Lymphocytes	11.6 ± 14.8
Lymphocytes T	7.9 ± 10.5
CD4	5.0 ± 7.5
CD8	3.0 ± 3.7
Ratio CD4/CD8	1.9 ± 1.5
Lymphocytes B	3.0 ± 5.0
Natural killer cells	1.1 ± 2.6
Neutrophils	13.2 ± 16.4
Eosinophiles	0.1 ± 0.4
Basophiles	0.0 ± 0.0
Monocytes	0.6 ± 1.3
Dendritic cells	0.1 ± 0.4
Fibroblasts	1.9 ± 3.7
Endothelium	1.9 ± 4.9
**Tumor cells (%)**	**70.4 ± 25.1**
Tumor cells (events)	66,300 ± 107,839
All cells (events)	113,762 ± 248,646
White blood cells [cells/µ] (by hematological analyzer)	3325 ± 4855.3

**Table 3:** Differences in the proportion of analyzed cells in lymph node aspirates (LNs) between patients with lung cancer non-small-cell lung cancer (NSCLC) and those with small-cell lung cancer (SCLC). Data expressed as medians (Q1–Q3). * indicates statistically significant *p*.

Leukocytes Subpopulation and Tumor Cells [% of All Cells]	NSCLC Median (Q1–Q3)	SCLC Median (Q1–Q3)	* p < 0.05 Mann–Whitney U Test
Lymphocytes	8.5 (7.1–32.4)	4.0 (1.0–8.4)	* *p* = 0.0249
Lymphocytes T	6.3 (3.3–20.5)	3.2 (0.8–6.4)	* *p* = 0.0296
CD4	3.5 (1.5–8.4)	1.2 (0.4–4.0)	*p* = 0.1041
CD8	3.2 (0.9–5.5)	1.4 (0.3–2.2)	* *p* = 0.0241
Ratio CD4/CD8	1.1 (0.8–1.9)	1.3 (1.0–2.3)	*p* = 0.3515
Lymphocytes B	1.8 (0.5–6.3)	0.6 (0.3–1.7)	* *p* = 0.0331
NK cells	0.4 (0.0–1.2)	0.2 (0.0–0.9)	*p* = 0.3990
Neutrophils	11.1 (2.0–22.2)	2.9 (0.8–17.8)	*p* = 0.4747
Eosinophiles	0.0 (0.0–0.0)	0.0 (0.0–0.0)	*p* = 0.8396
Basophiles	0.0 (0.0–0.0)	0.0 (0.0–0.0)	-
Monocytes	0.0 (0.0–0.0)	0.0 (0.0–1.4)	*p* = 0.4359
Dendritic cells	0.0 (0.0–0.0)	0.0 (0.0–0.0)	*p* = 0.5291
Fibroblasts	1.2 (0.2–4.8)	0.4 (0.1–1.0)	* *p* = 0.0313
Endothelium	0.6 (0.1–1.8)	0.1 (0.0–1.4)	*p* = 0.2025
Tumor cells (%)	62.5 (32.3–78.4)	88.5 (65.7–94.6)	* *p* = 0.0127
Tumor cells (events)	13,792 (3712–33,484)	50,829 (7018–136,923)	* *p* = 0.0350
All cells (events)	25,245 (7737–76,001)	59,925 (13045–144,124)	* *p* = 0.0185
White blood cells [cells/µ] (by hematological analyzer)	757 (333–1752)	2115 (765–6499)	* *p* = 0.0249

Abbreviations: NSCLC—non-small-cell lung cancer; SCLC—small-cell lung cancer.

**Table 4:** Differences in the proportion of analyzed antigens in lymph node aspirates (LNs) between patients with lung cancer non-small-cell lung cancer (NSCLC) and those with small-cell lung cancer (SCLC). Data expressed as medians (Q1–Q3). * indicates statistically significant *p*.

Scheme 1	NSCLC Median (Q1–Q3)	SCLC Median (Q1–Q3)	* p < 0.05 Mann–Whitney U Test
**[% of Tumor Cells]**			
EpCAM	70.1 (51.9–80.1)	87.5 (75.5–91.4)	* *p* = 0.0004
MUC-1	88.0 (82.7–94.3)	72.6 (59.9–79.0)	* *p* = 0.0018
TTF-1	77.0 (48.2–85.0)	78.5 (65.6–85.3)	*p* = 0.6621
Ki67	72.7 (57.1–90.9)	65.5 (54.2–77.8)	*p* = 0.1735
Cytokeratin	82.3 (43.9–89.9)	72.8 (15.1–89.0)	*p* = 0.3203
CD56	60.4 (35.6–67.3)	88.5 (61.9–96.7)	* *p* = 0.0112
CD38	8.0 (4.2–12.7)	2.4 (0.7–13.3)	*p* = 0.1194
HLA-DR	11.8 (5.7–59.4)	1.1 (0.6–3.7)	* *p* < 0.0001
HER-2	24.7 (16.8–50.0)	40.8 (5.6–64.0)	*p* = 0.7251
CD39	20.0 (10.1–30.9)	3.0 (0.9–10.5)	* *p* < 0.0001
CD73	25.8 (16.2–58.6)	18.1 (12.5–32.9)	*p* = 0.1950
CD90	9.8 (3.1–19.1)	26.3 (5.4–54.0)	*p* = 0.3660
CD184	16.5 (2.9–27.1)	35.1 (13.6–67.2)	* *p* = 0.0085
PD-L1	21.6 (8.3–45.1)	6.4 (0.3–13.4)	* *p* = 0.0046
PD-L2	58.7 (39.5–71.9)	26.7 (8.2–42.9)	* *p* < 0.0001
CTLA-4	3.2 (1.1–7.1)	21.4 (2.2–46.6)	* *p* = 0.0122
**[GMF of tumor cells]**			
EpCAM	4769.2 (2503.3–7279.3)	30,007.9 (9817.8–56,446.9)	* *p* < 0.0001
MUC-1	8965.3 (4320.0–21,780.5)	2412.0 (2072.7–4270.2)	* *p* = 0.0003
TTF-1	4260.8 (1969.7–6719.4)	3725.7 (2706.6–5928.6)	*p* = 0.9405
Ki67	1912.2 (1433.3–2748.2)	1657.1 (1429.8–1927.1)	*p* = 0.1735
Cytokeratin	3831.0 (1384.0–6446.8)	2545.1 (1041.4–7083.0)	*p* = 0.3203
CD56	3803.5 (2421.2–4995.8)	15,059.8 (52,360.0–30,566.3)	* *p* = 0.0111
CD38	1350.4 (809.5–1570.4)	1073.3 (555.2–1864.1)	*p* = 0.5866
HLA-DR	1690.0 (988.4–4748.0)	655.3 (401.0–809.0)	* *p* = 0.0008
HER	1373.0 (954.1–2796.2)	1945.3 (745.8–4348.7)	*p* = 0.6621
CD39	1825.1 (1380.9–2286.2)	927.4 (565.0–1406.9)	* *p* < 0.0001
CD73	1108.8 (826.1–3055.8)	919.7 (778.9–1099.0)	*p* = 0.0872
CD90	593.3 (192.6–924.9)	2851.4 (171.4–7822.3)	*p* = 0.1807
CD184	981.5 (171.3–2219.6)	3396.1 (1389.2–8300.6)	* *p* = 0.0014
PD-L1	837.8 (515.5–1109.7)	419.2 (265.7–506.4)	*p = 0.0045
PD-L2	967.0 (652.8–1352.5)	525.1 (376.4–667.4)	* *p* < 0.0001
CTLA-4	1340.1 (1001.6–1597.5)	2979.7 (885.6–8482.2)	*p = 0.0371

Abbreviations: NSCLC—non-small-cell lung cancer; SCLC—small-cell lung cancer; EpCAM—epithelial cell adhesion molecule; MUC-1—Mucin-1; TTF-1—thyroid transcription factor-1; HER-2—human epidermal growth factor receptor 2; PD-L1—Programmed death-ligand 1; PD-L2—Programmed death-ligand 2; CTLA-4—cytotoxic T cell antigen 4; GMF—geometric mean fluorescence.

**Table 5:** Differences in the proportion of hematological parameters in lymph node aspirates (LNs) between patients with non-small-cell lung cancer (NSCLC) and those with small-cell lung cancer (SCLC). Data expressed as medians (Q1–Q3). * indicates statistically significant *p*.

Hematological Parameters	NSCLC Median (Q1–Q3)	SCLC Median (Q1–Q3)	* p < 0.05 Mann–Whitney U Test
NE-SSC [ch] (or NE-GI)	150.6 (134.4–153.7)	119.1 (113.3–152.3)	* *p* = 0.0296
NE-SFL [ch]	45.9 (43.1–47.7)	48.1 (45.0–50.6)	*p* = 0.1358
NE-FSC [ch]	72.4 (48.4–81.9)	66.0 (53.3–86.7)	*p* = 0.8563
LY-X [ch]	83.9 (82.3–89.7)	95.0 (84.4–100.4)	* *p* = 0.0075
LY-Y [ch]	53.8 (42.7–65.0)	48.7 (44.8–57.6)	*p* = 0.3990

Abbreviations: NSCLC—non-small-cell lung cancer; SCLC—small-cell lung cancer; ch—channel unity; NE-SSC—neutrophil granularity intensity (neutrophil complexity); NE-SFL—neutrophil reactivity intensity (neutrophil fluorescence intensity); NE-FSC—size or volume of neutrophils; LY-X—lymphocytes complexity; LY-Y—lymphocytes fluorescence; ch—channel.

## Data Availability

The data presented in this study are available in this article.

## Supplementary Materials

The following supporting information can be downloaded at: https://www.mdpi.com/article/10.3390/cancers17030431/s1, Figure S1: Representative flow cytometry gating strategy of lymph node aspirates with antibodies specific for cells of hematopoietic and non-hematopoietic origin. FSC-A vs. FSC-H plot: For removing clumps and debris, CD45 vs. SSC-A plot: Indication of lymphocytes based on their CD45+ SSC-A+dim characteristics. Next gating strategy for lymphocyte subpopulation: CD3 vs. CD19 plot: T lymphocytes with CD3+ antigen expression (CD45+ CD3+ SSC-A+dim characteristic, green) and B lymphocytes with CD19+ antigen expression (CD45+ CD19+ SSC-A+dim characteristic, pink), CD4 vs. CD8 plot: T lymphocyte subsets: CD4+ (CD45+ CD3+ SSC-A+dim CD4+ characteristic) and CD8+ (CD45+ CD3+ SSC-A+dim CD8+ characteristic) based on their CD4/CD8 expression. CD3 vs. CD16 plot: NK cells (yellow) with CD16+ antigen expression and no expression of CD3 antigen (CD45+ CD3- SSC-A+bright CD16+ characteristic). Neutrophil gating strategy: SSC-A vs. CD16 plot: Selection of neutrophils based on their CD16+ and SSC-A high properties and CD45 positive (CD45+ CD3- SSC-A+bright CD16+, navy). Monocyte gating strategy: SSC-A vs. CD64 plot and HLA-DR plots: Selection of monocytes based on their CD45+ CD64+ and HLA-DR+ properties (CD45+ CD64+ HLA-DR+ SSC-A+ characteristic, turquoise). FMA vs. SSC-A plot: Selected of fibroblasts (CD45+dim FMA+ SCC-A+ characteristci, brown), CD146 vs. EpCAM plot: Selected endothelium (CD45- CD146+ SSC-A+ characteristic, purple), CD146 vs. EpCAM and FMA vs. EpCAM and SSC-A vs. EpCAM: probably tumor cells (CD45- EpCAM+ SSC-A+brigh CD146- FMA-, blue). Table S1: The characteristics of the investigated group.
