# Supplementary material for: The Detection of Lung Cancer Cell Profiles in Mediastinal Lymph Nodes Using a Hematological Analyzer and Flow Cytometry Method
Source: Cancers (Basel). 2025 Jan 27;17(3):431. doi: 10.3390/cancers17030431 (PMC11816154; doi:10.3390/cancers17030431)
Supplement: Supplementary file 1 [file cancers-17-00431-s001.zip › cancers-3407096-supplementary.pdf]

Supplementary Materials:

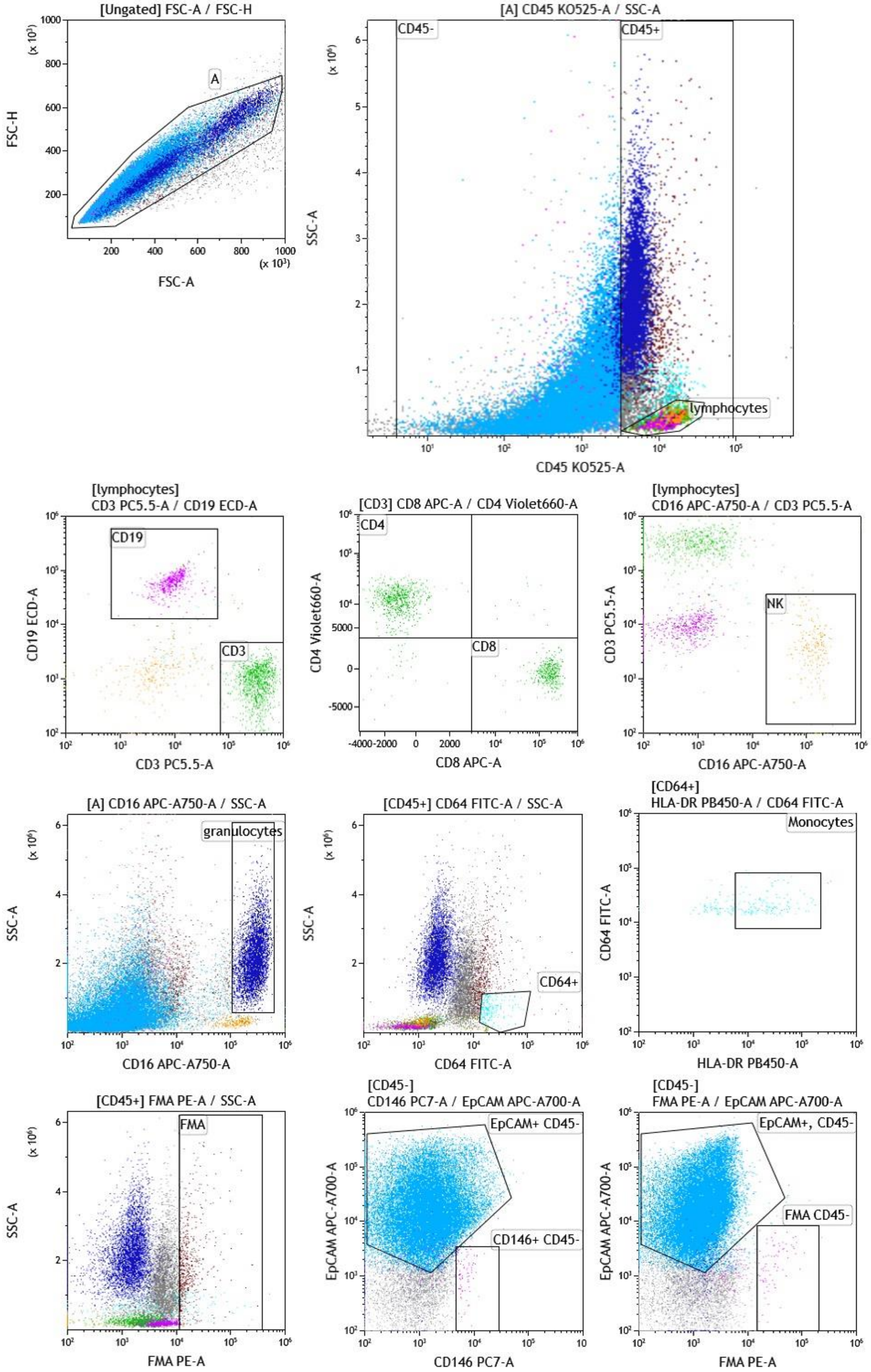

Figure S1. Representative flow cytometry gating strategy of lymph node aspirates with antibodies specific for cells of hematopoietic and non-hematopoietic origin. FSC-A vs. FSC-H plot: For removing clumps and debris, CD45 vs. SSC-A plot: Indication of lymphocytes based on their CD45+ SSC-A+dim characteristics. Next gating strategy for lymphocyte subpopulation: CD3 vs. CD19 plot: T lymphocytes with CD3+ antigen expression (CD45+ CD3+ SSC-A+dim characteristic, green) and B lymphocytes with CD19+ antigen expression (CD45+ CD19+ SSC-A+dim characteristic, pink), CD4 vs. CD8 plot: T lymphocyte subsets: CD4+ (CD45+ CD3+ SSC-A+dim CD4+ characteristic) and CD8+ (CD45+ CD3+ SSC-A+dim CD8+ characteristic) based on their CD4/CD8 expression. CD3 vs. CD16 plot: NK cells (yellow) with CD16+ antigen expression and no expression of CD3 antigen (CD45+ CD3- SSC-A+bright CD16+ characteristic). Neutrophil gating strategy: SSC-A vs. CD16 plot: Selection of neutrophils based on their CD16+ and SSC-A high properties and CD45 positive (CD45+ CD3- SSC-A+bright CD16+, navy). Monocyte gating strategy: SSC-A vs. CD64 plot and HLA-DR plots: Selection of monocytes based on their CD45+ CD64+ and HLA-DR+ properties (CD45+ CD64+ HLA-DR+ SSC-A+ characteristic, turquoise). FMA vs. SSC-A plot: Selected of fibroblasts (CD45+dim FMA+ SCC-A+ characteristci, brown), CD146 vs. EpCAM plot: Selected endothelium (CD45- CD146+ SSC-A+ characteristic, purple), CD146 vs. EpCAM and FMA vs. EpCAM and SSC-A vs. EpCAM: probably tumor cells (CD45- EpCAM+ SSC-A+brigh CD146- FMA-, blue).

Table S1. The characteristics of the investigated group.

Abbreviation: M: male; F: female; LNs: lymph nodes; SCLC: smal cell carcinoma; SQCLC: squamous cell lung carcinoma; ADC: lung adenocarcinoma; LCC: Large cell carcinoma; NOS: not otherwise specified; PD-L1: Programmed death-ligand 1; TTF1: Thyroid transcription factor 1; CK7: Cytokeratin 7; CK5/6: Cytokeratin 5/6; CKAE1/AE3: Cytokeratin AE1 / AE3; Cytokeratin AE1 / AE3; MUC1: mucin; EMA: Epithelial membrane antigen

Case	Aga	Sex	LNs	Histological subtype	PD-L1 (%)	Ki67 (%)	p63	p40	p16	TTF1	CK7	CK5/6	CKAE1/AE3	synaptophysin	chromogranin	Mucicarmine	MUC1	MUC2	MUC5	EMA
1	76	f	4L	SCLC		100				+			+	+	-					
2	60	m	7	SCLC							+		+	-	-					+
3	80	m	4L	SCLC		90				-			+	+	+					
4	74	F	11R	SCC			+			-		+								
5	74	m	4R	ADC	95		+			+										
6	75	m	4R	SCLC		75				+			+	+	+					
7	70	f	7	SCLC		80				+			+	+	-					
8	73	f	11L	SQCLC	0		+			-	+	+								
9	67	m	4R	SQCLC	10	80	+			-	+	+			-					
10	77	f	4R	ADC	1		+			+	+									
11	68	m	11L	SCLC		80				+			+	+	+					
12	61	f	4L	SCLC		70				+			+	+	+					
13	75	m	7	ADC	5		-			+	+	-								
14	49	f	7	SCLC		40				+			+	+	+					
15	68	f	4R	SCLC						-			+	+	+					
16	63	f	tumor nodal mass	SCLC																
17	76	f	tumor nodal mass	SCLC																
18	68	f	4R	LCC	70		+			-	+	-	+	-	-					
19	62	f	tumor nodal mass	SCLC																
20	53	f	tumor nodal mass	SQCLC				+	+	-										
21	69	m	7	ADC						+							-/+	-	+	
22	55	f	tumor nodal mass	SCLC																
23	50	m	11L	ADC				-		+	+			-						
24	61	f	11R, 7	NOS				-		-	+									

25	78	m	tumor nodal mass	ADC				-		+						-				
26	72	f	4R	SCLC		90		-		+				+	+					
27	50	f	4L	SCLC		90		-		-			+	+						
28	68	M	10L	SCLC		80		-		+			+	+						
29	74	f	4R	SCLC																
30	71	f	tumor nodal mass	SCLC											-					
31	72	M	4R	SCLC																
32	59	M	4L	SCLC																
33	58	M	4R	SQCLC																
34	87	M	7	ADC				-		+										
35	72	M	tumor nodal mass	SCLC		70		+		-			+							
36	71	f	tumor nodal mass	ADC				-		-						+				
37	65	f	tumor nodal mass	NOS				-		+										
38	66	f	11L	NOS				+		-				+	+					
39	76	M	7	NOS				-		-										
40	66	M	7	SCLC																
41	74	M	tumor nodal mass	SQCLC																
42	69	f	4R	ADC				-		+										
43	69	f	7	SCLC																
44	72	f	4R	SCLC				"-		-			+/-	+						
45	80	m	4L	SCLC		90				+			+	+						
46	61	m	4L	SCLC																
47	69	m	tumor nodal mass	SCLC																
